# Prospective Optimization with Limited Resources

**DOI:** 10.1371/journal.pcbi.1004501

**Published:** 2015-09-14

**Authors:** Joseph Snider, Dongpyo Lee, Howard Poizner, Sergei Gepshtein

**Affiliations:** 1 Institute for Neural Computation, University of California at San Diego, La Jolla, California, United States of America; 2 Graduate Program in Neurosciences, University of California at San Diego, La Jolla, California, United States of America; 3 Systems Neurobiology Laboratories, Salk Institute for Biological Studies, La Jolla, California, United States of America; University of Minnesota, UNITED STATES

## Abstract

The future is uncertain because some forthcoming events are unpredictable and also because our ability to foresee the myriad consequences of our own actions is limited. Here we studied how humans select actions under such extrinsic and intrinsic uncertainty, in view of an exponentially expanding number of prospects on a branching multivalued visual stimulus. A triangular grid of disks of different sizes scrolled down a touchscreen at a variable speed. The larger disks represented larger rewards. The task was to maximize the cumulative reward by touching one disk at a time in a rapid sequence, forming an upward path across the grid, while every step along the path constrained the part of the grid accessible in the future. This task captured some of the complexity of natural behavior in the risky and dynamic world, where ongoing decisions alter the landscape of future rewards. By comparing human behavior with behavior of ideal actors, we identified the strategies used by humans in terms of how far into the future they looked (their “depth of computation”) and how often they attempted to incorporate new information about the future rewards (their “recalculation period”). We found that, for a given task difficulty, humans traded off their depth of computation for the recalculation period. The form of this tradeoff was consistent with a complete, brute-force exploration of all possible paths up to a resource-limited finite depth. A step-by-step analysis of the human behavior revealed that participants took into account very fine distinctions between the future rewards and that they abstained from some simple heuristics in assessment of the alternative paths, such as seeking only the largest disks or avoiding the smaller disks. The participants preferred to reduce their depth of computation or increase the recalculation period rather than sacrifice the precision of computation.

## Author Summary

We investigated the human ability to organize behavior prospectively, for multiple future steps in risky, dynamic environments. In a setting that resembled a video game, participants selected the most rewarding paths traversing a triangular lattice of disks of different sizes, while the lattice scrolled down a touchscreen at a constant speed. Disk sizes represented the rewards; missing a disk incurred a penalty. Every choice excluded a number of the disks accessible in the future, encouraging subjects to examine prospective paths as far into the future as they could. In contrast to previous evidence that humans tend to reduce the computational difficulty of decision making by means of simplifying heuristics, our participants appeared to perform an exhaustive computation of all possible future scenarios within a horizon limited by a fixed number of computations. Under increasing time pressure, participants either reduced the computational horizon or recalculated the expected rewards less frequently, revealing a resource-limited ability for rapid detailed computation of prospective actions. To perform such intensive computations, participants could take advantage of the massively parallel neural architecture of the visual system allowing one to concurrently process information from multiple retinal locations.

## Introduction

Your actions in the present depend on how you reckon the future: which ramifications of present actions you consider, and how far into the future you can trace these ramifications as their number and complexity increase with the scope of prospective outlook. Such prospective computations are complicated by two kinds of uncertainty: extrinsic, which is independent of the planner, and intrinsic, which depends on the planner’s ability to perform the computation. In other words, even when the information needed for the prospective computation is explicit and unambiguous, planner’s limited computational powers restrict the depth and quality of prospection.

**Fig 1:**
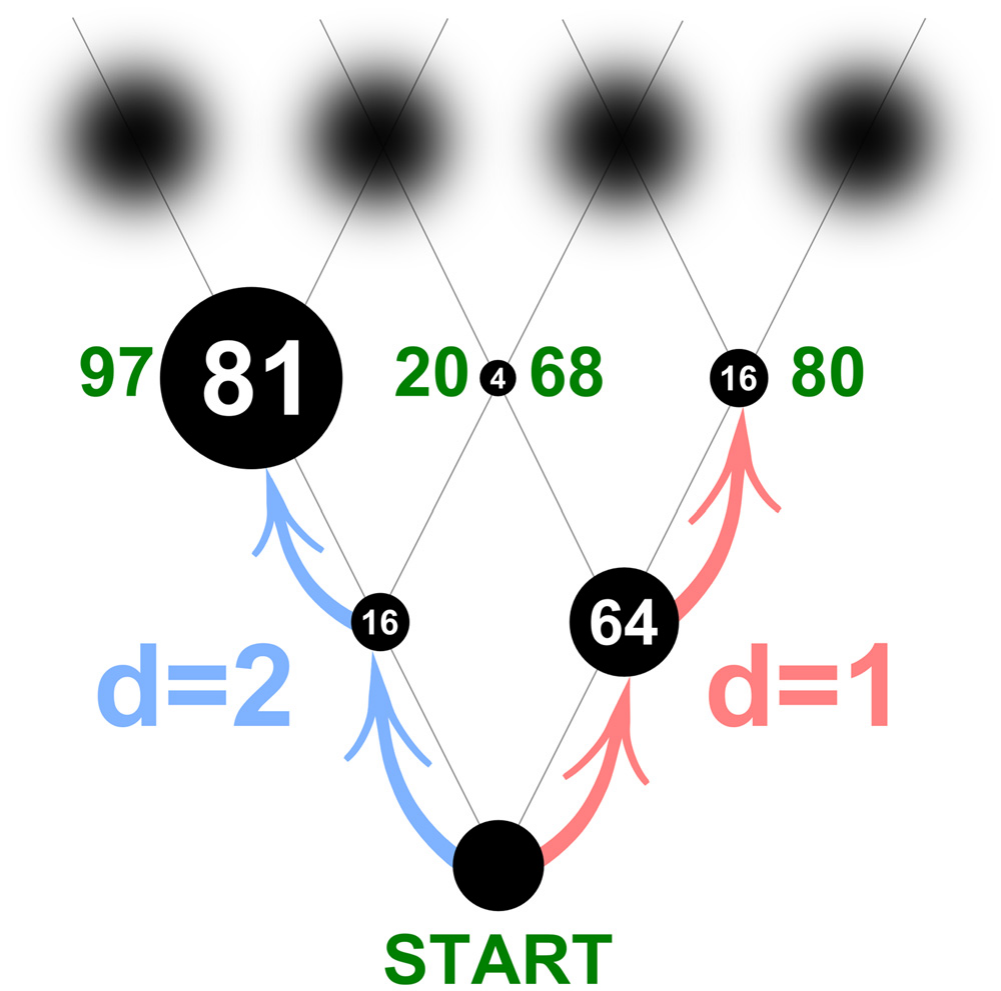
Binary decision tree. Moving up from the START disk, actors make sequential binary choices between the rewards represented by disk sizes (displayed inside the disks). The task is binary in that the actor has to choose between two alternatives on every step: upward left or upward right. Actors characterized by different depths of computation *d* will choose different paths. The short-sighted actor with depth *d* = 1 (red) will collect 80 points (displayed in green in the third row). The more far-sighted actor with depth *d* = 2 (blue) will collect 97 points.

For example, consider the binary “decision tree” in Fig 1. Suppose a short-sighted actor Arthur discounts the future and plans actions for one step at a time, i.e., his depth of computation, *d*, is one row. From the bottom disk of the decision tree, Arthur will step first to the right, choosing the reward of 64 points, and then to the right again, choosing 16 points and earning 80 points in total. Now consider another actor Merlin who values the future more than Arthur and computes rewards for two rows ahead (*d* = 2). Merlin can see the large reward of 81 points in the third row, and so he will first step to the left, losing 48 points (64-16) in the short run, then step to the left again, to collect the winning 97 points in total by the time he reaches the third row. Notably, the large reward of 81 points in the third row will be inaccessible to the short-sighted Arthur after he has made the first step. The two actors have the same information in front of them, but their actions differ because of their internal limitations: Arthur has a shorter outlook and thus a greater intrinsic uncertainty about things to come than Merlin.

Looking farther ahead comes at a price because the world may change while the actors execute their plans, independent of their choices. For instance, a new high-value disk could appear in the top row (e.g., second from the right) and give an advantage to the short-sighted actor, Arthur, who will be able to reach that target. To avoid being trapped in a fixed plan, the longer-sighted Merlin may recalculate his plan at every step, flexibly incorporating new information. This way, extrinsic uncertainty prompts actors to be flexible, pressing against dogmatic adherence to pre-computed plans.

An additional drawback to looking far ahead is the larger amount of computation required to evaluate the alternative paths. Even on the small decision tree in Fig 1, Merlin would have to evaluate twice as many paths as Arthur (assuming he did an exhaustive search for the best path). Yet, the actors could employ algorithms with significant computational savings. For example, they could ignore the small rewards and just seek out the largest ones. This heuristic approach is an example of the strategy (sometimes called “satisficing” [1, 2] or “pruning” [3–5]) in which the actors are content with a reduced sampling of the search space, even at the cost of a lower reward. Alternatively, actors could use highly efficient search strategies (as we show below) and find the global optimum at significant savings to the amount of computation. Previous studies showed that humans can, indeed, use optimal algorithms to solve such problems [6–9].

Previous research of prospective planning has approached this problem from a number of directions, of which the most notable are reinforcement learning and decision theory. The literature on reinforcement learning has offered a rich repertoire of biologically plausible solutions [4, 10–13] to the general problem of sequential decision-making and dynamic programming [14, 15]. The central question addressed by this literature is how biological organisms learn efficient strategies for multi-step behavior in complex uncertain environments: how the rewards that may be delayed or arrive on a random schedule can shape decision policies that lead to successful behavior.

Studies of prospective computations in the decision-theoretic framework are more recent and they have a different focus. They tend to concentrate on the structure of individual decisions rather than on the method of learning efficient strategies, focusing in particular on how planners combine information about the expected risks and uncertainty for decision making (reviewed in [16, 17]). Studies in this framework have demonstrated that humans plan their active behavior using representations of their intrinsic uncertainty: the uncertainty about outcomes of their own actions [6, 18, 19]. These representations are flexible: they are readily updated when the uncertainty appears to change [20, 21]. Also, different relevant representations are evoked in preparation for actions characterized by different uncertainties [22]. But the actions investigated in the decision-theoretic framework have been temporally local; they involved only one or two steps at a time.

**Fig 2:**
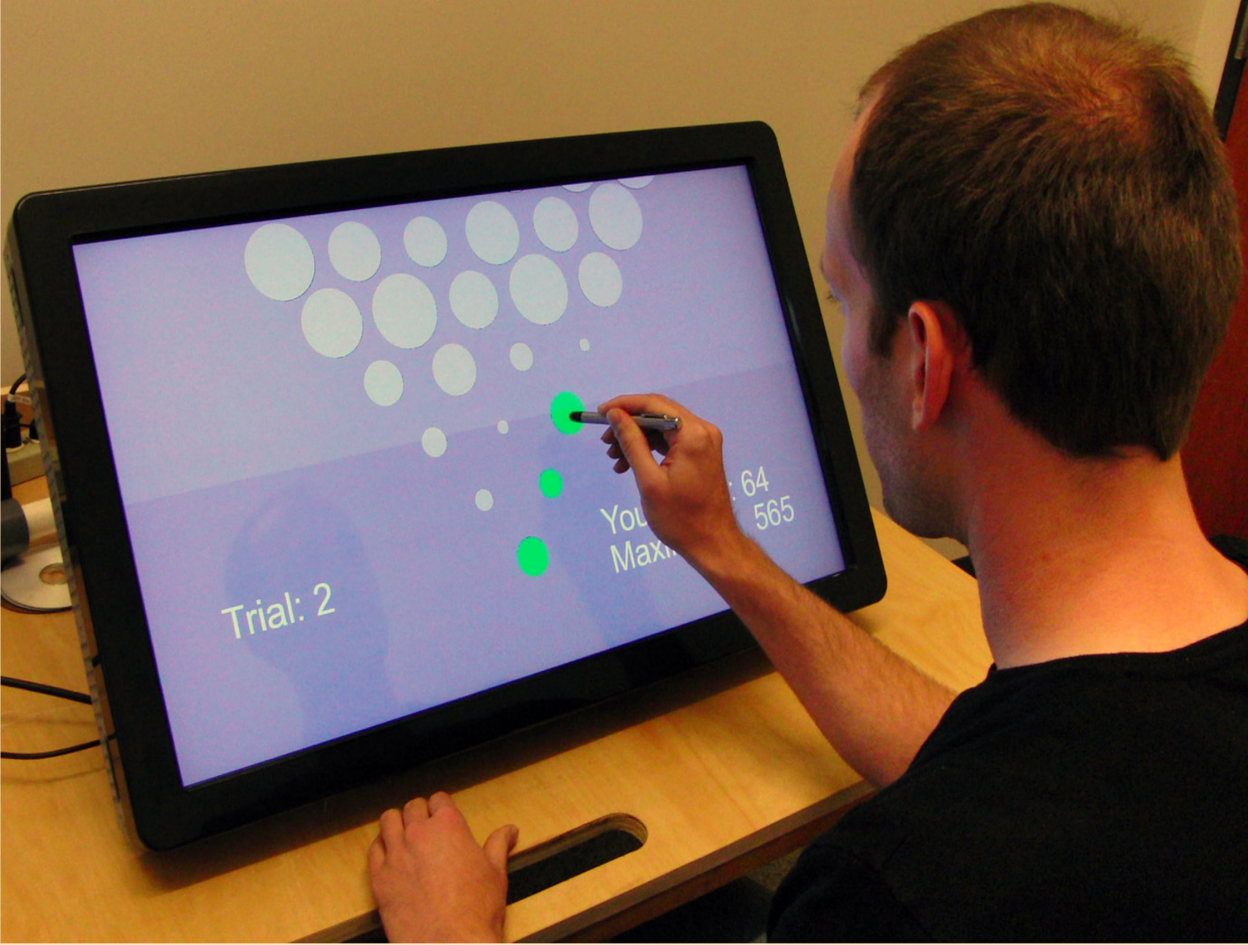
Participant performing the task. The stimulus is a triangular lattice of disks of different sizes scrolling down on a touch-sensitive screen. The task is to collect maximal score by touching the disks: the larger the disk the larger the score. On every step, the participant makes a binary choice; only one of the two adjacent disks may be touched above the just-touched disk.

Here we study sequential decision-making in actions that involve multiple steps, using a task anticipated in Fig 1 and illustrated in Fig 2. We follow the decision-theoretic tradition because we investigate the scope and structure of prospective computations rather than the methods for acquiring prospective competency [13]. We ask human actors to execute rapid sequences of actions on a dynamic decision tree rendered as a visual stimulus, in which the values of forthcoming stimuli are given explicitly and thus do not need to be learned. We infer human depth of computation and the rate of recomputing the course of action by finding the ideal actors whose choices are most similar to the human choices.

We find that humans behave as if they allocate limited computational resources across the field of future possibilities, sacrificing larger depths of computation for more frequent recalculation, and *vice versa*. As expected, a reduced difficulty allows human actors to expand their depth of planning, and also use information within the scope of planning more efficiently. Surprisingly, our results indicate that humans do not use some obvious decision heuristics. Even under high pressure, they do not employ such simplifying strategies as seeking larger rewards or avoiding smaller rewards. Instead, they appear to use all the information available within a limited horizon to exhaustively search for the most rewarding path through the stimulus.

## Results

### Multi-step decision task

Participants sat in front of a touchscreen on which a triangular lattice of disks of different sizes (the “stimulus”) scrolled down at a variable speed (Fig 2). Presentation of one complete stimulus constituted one “trial.” The task was a forced binary choice; participants were instructed to maximize the cumulative score by touching one disk at a time in a rapid sequence, starting from the bottom row (which contained one disk) and moving up by touching one of the two disks above the one just touched. Touching a disk incurred a reward proportional to disk area. Missing a disk incurred a penalty, while timeouts and incorrect steps (skipping a row or touching a disk other than the two allowed) terminated the current trial and initiated the next trial with a different stimulus. There were four experimental conditions: two scrolling speeds and two penalties for missing the disks, detailed in *Methods*.

### Informative stimuli

**Fig 3:**
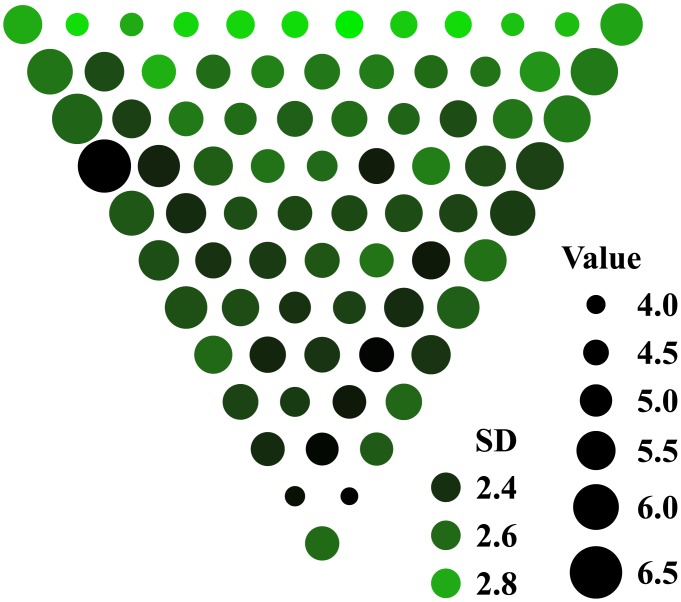
Average stimulus. Stimuli were selected to maximize the ability to infer planning strategies from the observed behavior. The average stimulus is colored by the variability of disk size. Disk size represents the average size across all trials. Disk variability is the highest on the last row. “SD” stands for standard deviation. Four examples of the actual stimuli are shown in Fig 4.

**Fig 4:**
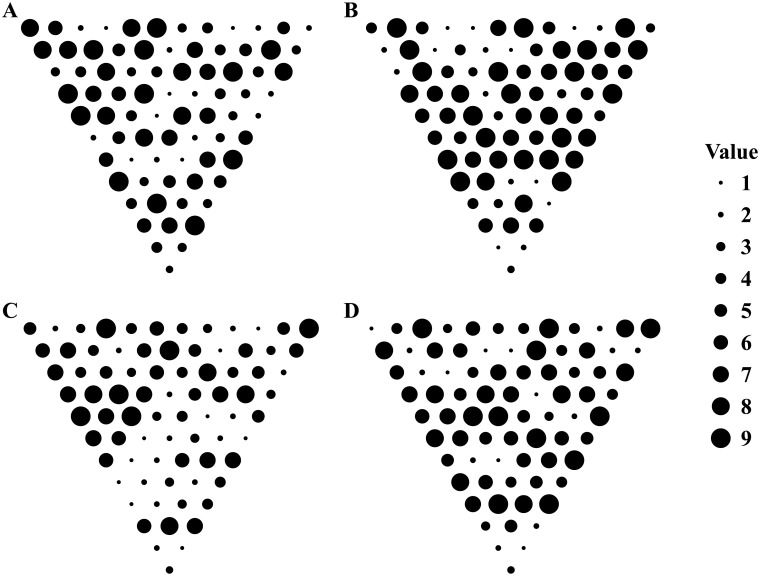
(A–D) Four instances of the maximally-informative stimuli. The stimuli were designed to maximize our ability to infer planning strategies from the observed behavior. Disk size represents its value.

To better identify actors’ strategies from the paths selected by humans, we designed the stimuli such as to minimize the overlap between the paths preferred by the different strategies. For every stimulus, the algorithm started with a random assignment of disk values and then randomly changed one value on every iteration. The total number of strategies leading to the overlapping paths on every disk was minimized using a Monte Carlo process described in *Methods*. Following multiple iterations of this process, the resulting stimuli had a non-uniform distribution of disk values summarized in Fig 3. (Several examples of individual stimuli are illustrated in Fig 4).

**Fig 5:**
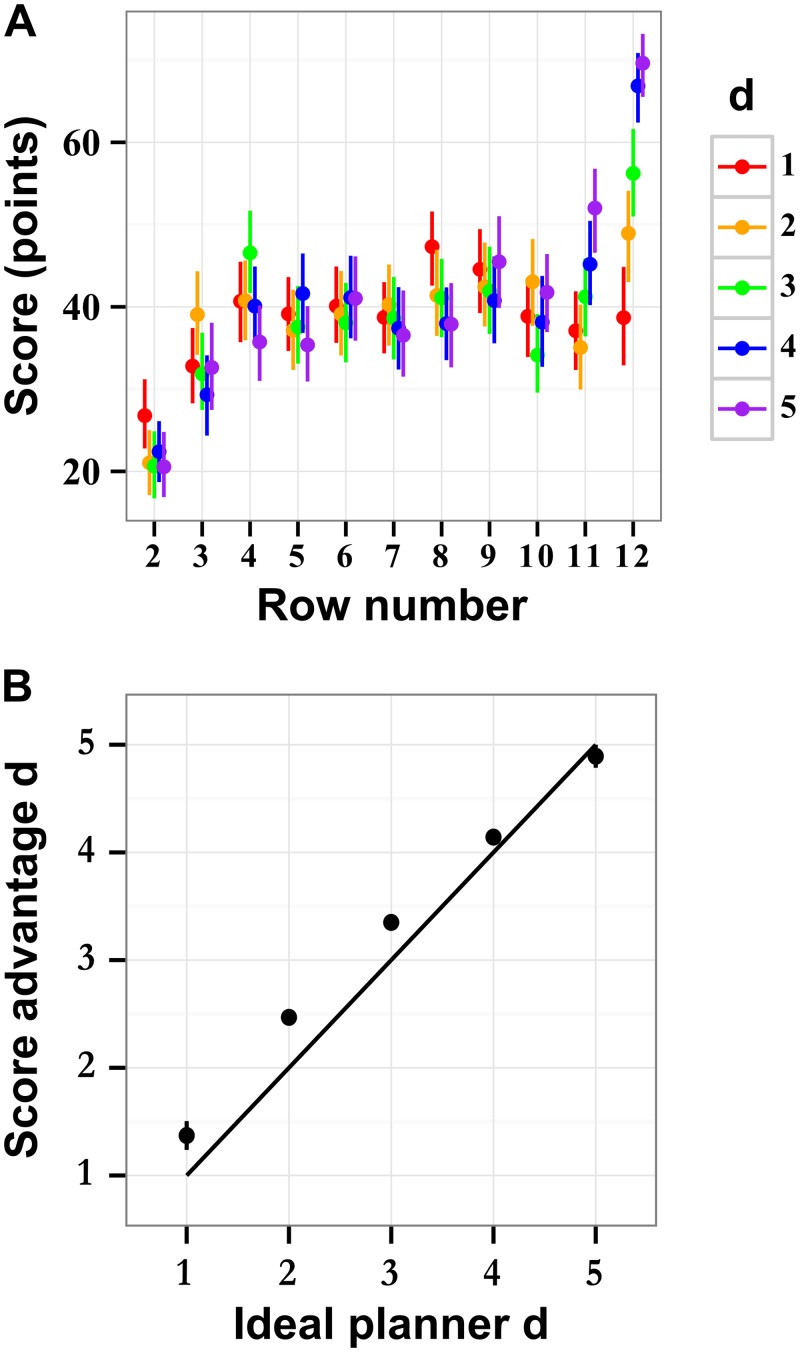
Effect of depth of computation on the score of ideal actor. (A) The scores collected by ideal actors with different fixed depths of computation *d* across stimulus rows. On the first rows, short-depth actors have an advantage over long-depth actors, which is reversed on the last rows. The largest gains are put off until the last rows. (B) A comparison of the average *d* from ideal actors with variable *d* to the best matching single *d* policy. The best match is chosen to minimize the score advantage on the last 3 rows. The solid line indicates perfect recovery. There is some systematic deviation, but the score matching algorithm recovers an approximation of the average *d*. All results are shown for a fixed recalculation period *r* = 1, points indicate mean value, and the error lines are bootstrapped 95% confidence intervals.

The diagnostic stimuli had two characteristic features. First, larger rewards appeared at the stimulus edges. Second, disk variability increased towards the last row. That is, the selected stimuli enticed actors toward stimulus edges by means of moderate rewards, but then pulled them back toward the middle of the stimulus by means of the large rewards on the last stimulus row. The expected gains grew towards the last row of the stimulus (Fig 5A), encouraging the actors to accumulate ongoing moderate rewards while looking ahead for the larger rewards at the stimulus end. The largest gains were only accessible to the actors with a large depth of computation *d*. The score collected on the last rows increased monotonically with depth of computation, *d*.

Actors were extensively trained on the task. Two separate sessions on different days of all four task conditions were completed by the actors to eliminate learning effects in the subsequent testing. The data were collected in ten consecutive blocks of ten trials each, for each experimental condition. To verify that there was no learning or fatigue over the course of the session, we fit a linear mixed model to the scores won by the participants on each stimulus (normalized to the maximum score on that stimulus) including effects of block, speed, and penalty. There were no significant effects of block (*p* > 0.35), and only a main effect of speed (*χ* ^2^ (1) = 7.9, *p* = 0.005), while the scores were 3.8 ± 0.4% higher for the slow than fast speeds.

### Identifying human strategies

#### Markov processes

Researchers of sequential decision making often assume that agent’s choices can be modeled using the framework of Markov decision process (MDP) [14]. Generalizing MDP to include probabilistic information leads to the partially observable Markov decision process (POMDP) that learns to make the choices that maximize total reward based on such unreliable information [23, 24]. In our task, information about the upcoming disks is incomplete: either because the disks are yet invisible or because their distance from the current disk is larger than the depth of computation *d*. A POMDP framework can be deployed to determine the optimal depths of computation given specific assumptions about 1) the desirability of large depths of computation, 2) a discount factor that devalues delayed rewards for the allowed actions, and 3) a model that maps observable differences in reward to actions. The large number of parameters required to specify a POMDP model (described in section *Comparison with partially observable Markov decision process* below) allows for a remarkable flexibility in the design of artificial intelligences capable of performing tasks of this nature. At the same time, the large number of parameters leads to significant difficulties in interpreting human data, known as the “inverse reinforcement learning” problem [25–27].

#### Ideal planners

Here we consider two alternative approaches that require a smaller number of assumptions about human strategies. In both, human behavior is compared with behavior of models called “ideal planners” whose choices are defined by a small number of parameters. Using the ideal planners, we characterize the sequential choices in terms of a “strategy” defined by two parameters, depth of computation *d* and recalculation period *r*. Depth of computation is the number of forthcoming steps (rows of the stimulus) for which the actor computes the expected reward. Recalculation period is the number of steps before the actor performs a new computation. (Note that *r* ≤ *d* since actors must recalculate when they reach the end of the pre-computed part of the stimulus). An actor capable of a large depth of computation is often compelled to sacrifice an immediate reward for the sake of a large future reward.

#### Identification by total scores

First, we compare human total scores with total scores of such ideal planers. The total score in our task depends on the actor’s ability to compute the expected rewards for multiple alternative courses of action and select the most promising alternative (Fig 1). This way we identify simple strategies consistent with human behavior under different magnitudes of penalty and time pressure. Concentrating on total-score comparisons does not allow one to distinguish the strategies underlying step-by-step choices by the participants. We demonstrate, however, that the total-score analysis leads to unambiguous results that make the loss of resolution worthwhile.

#### Identification by individual moves

Second, beyond the omnibus characterization of behavior using the cumulative estimates of behavior over multiple rows of the stimulus, we focus the analysis on individual rows. We compare the sequential choices by humans with the sequential choices selected by ideal planners. The comparison of step-by-step actions is most reliable in case of unique, unambiguous overlaps of the moves by human and ideal planners, which happen whenever a human action is identical to an action by just one ideal planner from a family of ideal planners. Thanks to the preselection of informative stimuli, such unambiguous overlaps occur in a significant number of cases, allowing us to improve fidelity of identifying human strategies. We also analyze human behavior in terms of reaction times of individual moves, assuming that the longer reaction times indicate longer planning and thus larger depths of computation. We find that these approaches yield consistent results and help to reveal human prospective strategies.

### Analysis of scores

We compared the overall score gained by every participant to the scores of various ideal actors. We computed *score advantage*, the difference of human and ideal scores:

$$\begin{array}{c} A\left(r,d\right)=S_{\text{h}}-S_{\text{i}}\left(r,d\right), \end{array}$$ (1)

where *S*_h_ is the score collected by the human actor and *S*_i_ is the score collected by an ideal actor that uses strategy (*r*, *d*). Participants had a positive or negative score advantage *A* in comparison with the ideal actors characterized, respectively, by low or high magnitudes of depth of computation *d* (Fig 5).

**Fig 6:**
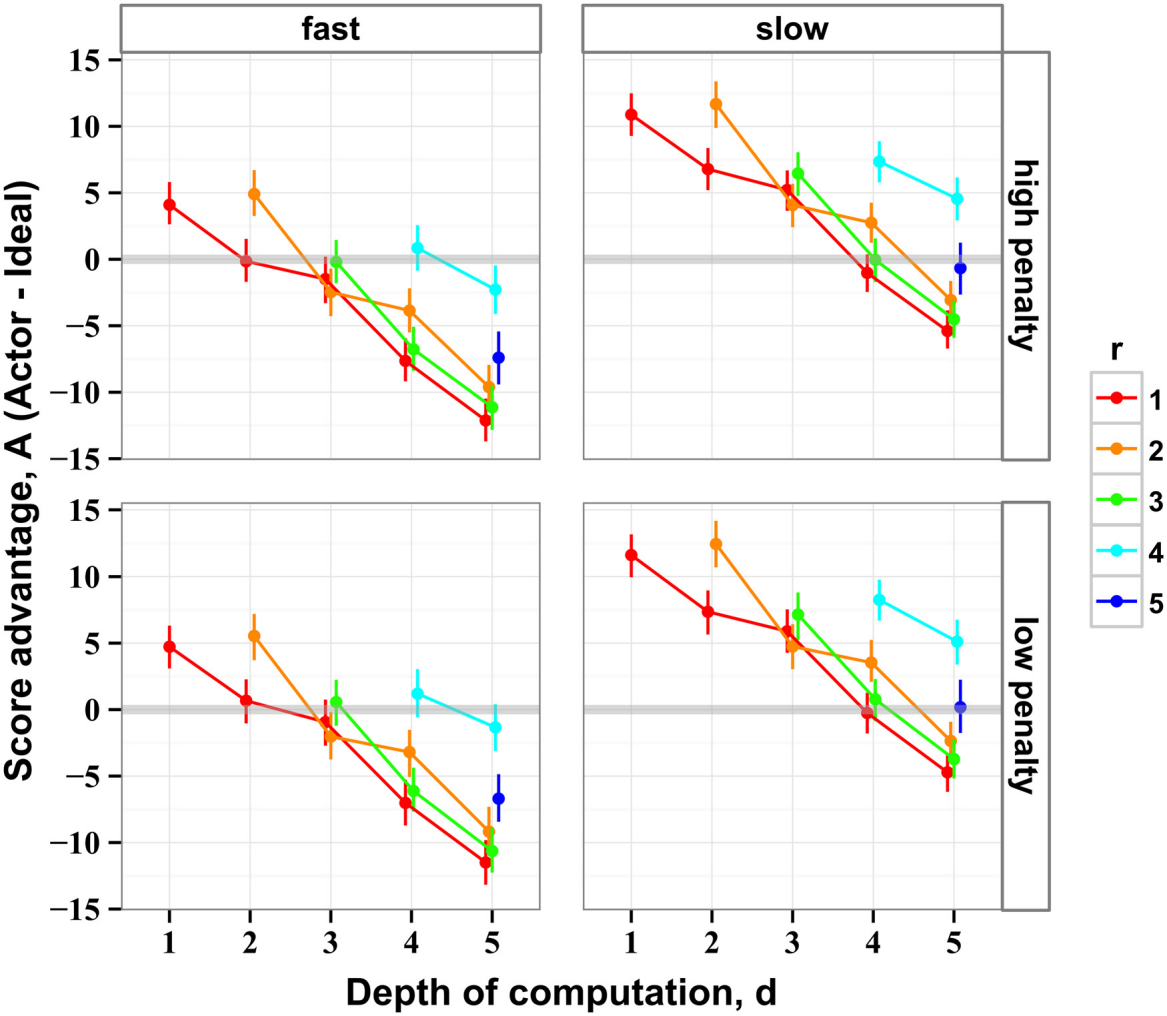
Score advantage of humans over ideal actors. Score advantage is the difference of the scores collected by human and ideal actors, the latter characterized by two fixed parameters: depth of computation *d* and recalculation period *r*, (Eq 1). The difference was accumulated over the last three stimulus rows where the differences between the human and ideal scores were the largest. The zero-crossings (where human behavior was most similar to that of ideal actors) were used to estimate the strategy used by humans. The data are shown for all experimental conditions: two stimulus speeds (fast and slow) and two magnitudes of penalty (high and low). The points are the means and the error bars are the 95% confidence intervals.

Equal scores of human and ideal actors indicated equally efficient strategies. We therefore used zero-crossings of score advantage as an estimate of the strategy that humans used to perform the task. In Fig 6, score advantage of human actors is plotted as a function of strategy (*r*, *d*) of ideal actors for every tested condition of stimulus speed and penalty. In all conditions, and for all values of *r*, human score advantage decreased linearly as a function of *d*.

Ideal actors were used to approximate the potential strategies used by humans. First, we assumed that humans used a constant strategy throughout the stimulus. (We relax this assumption below). To validate the method of measuring human performance by means of score advantage, we used an ideal actor with a random policy. At each row of the stimulus, the planner chose a random depth of computation, *d*, calculated the best (maximally scoring) path given the *d*, and took one step of the plan (*r* = 1; see Methods for details of the validation algorithm). The result in Fig 5B reveals a linear relationship between the average magnitudes of *d* of the ideal actor and the magnitude estimated by the score advantage method. In spite of a small systematic deviation, the magnitudes of *d* derived from score advantage were within ∼ 0.1 row of the correct mean depth.

We analyzed the data using a linear mixed model, allowing for potentially different slopes and offsets of score advantage as a function of (*r*, *d*) for different conditions. We found that both *d* and *r* were significant predictors of score advantage (*d*: *χ* ^2^ (1) = 40.6, *p* = 1.9 × 10^−10^ and *r*: *χ* ^2^ (1) = 28.3, *p* = 1.0 × 10^−7^). Stimulus speed also affected score advantage (*χ* ^2^ (1) = 28.3, *p* = 1.0 × 10^−7^), but stimulus penalty had no effect. We found no interactions of the slope and either stimulus speed or stimulus penalty.

**Fig 7:**
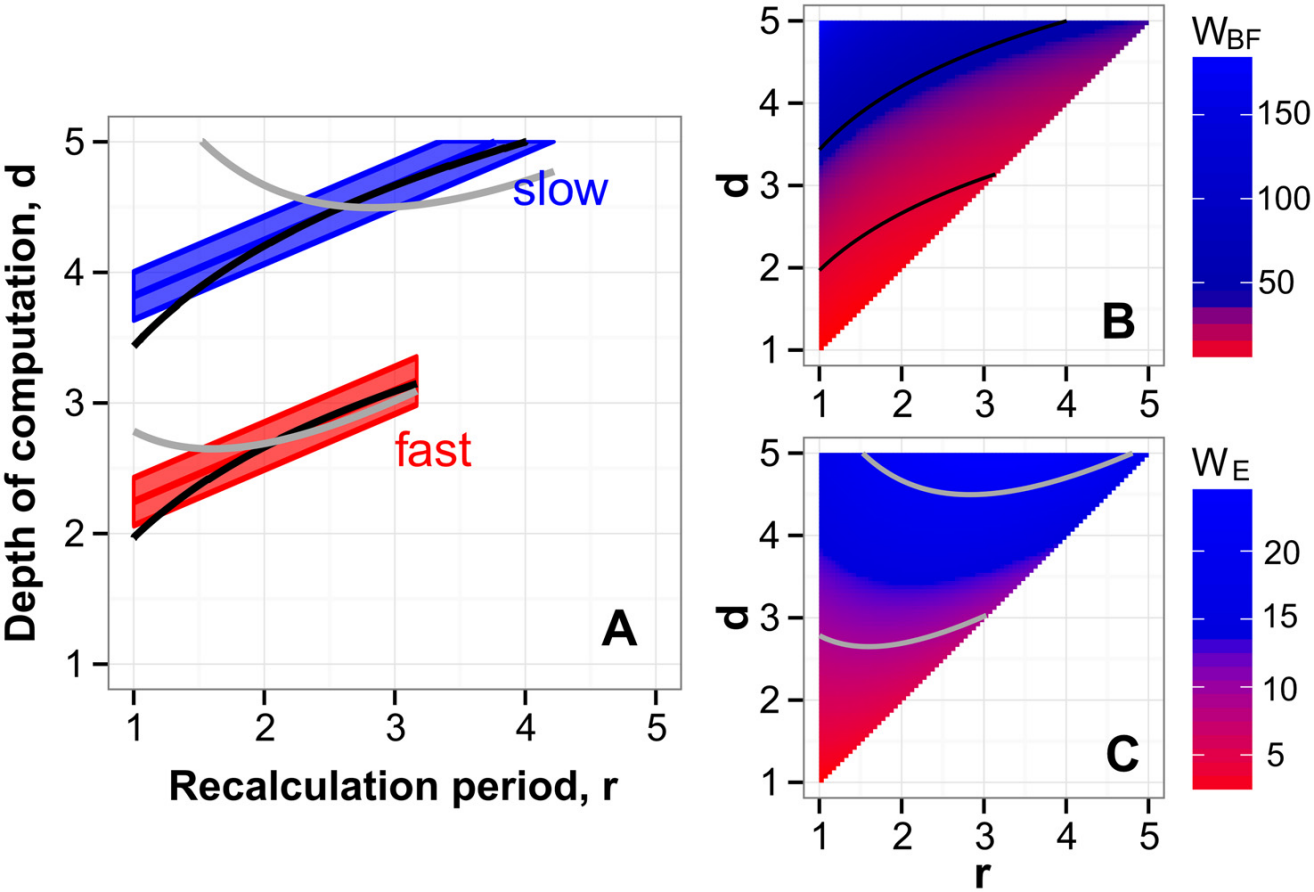
Strategies consistent with behavior of participants. (A) The shaded regions represent one standard error of the estimates of human strategies (*r*, *d*). The estimated values of *d* for a given *r* increased as stimulus speed decreased, indicating that the actors looked farther ahead when they had more time. The positive slopes of the contours indicate a tradeoff between looking farther ahead (high *d*) and recomputing the plan more often (small *r*). (B) A map of brute-force workload *W*_BF_: the number of summations required to calculate path value by the inefficient algorithm in Eq 2, plotted for all strategies (*r*, *d*). The black curves are the contours of constant *W*_BF_. (C) A map of workload *W*_E_ calculated using the efficient algorithm in Eq 3. The gray curves are the contours of constant *W*_E_. The black curves in panel A are the contours of constant workload *W*_BF_ from panel B that matched the estimated actor strategies. The gray curves are the contours of constant workload *W*_E_ from panel C which were the closest to the observed human strategies, but which could not match human data as well as the contours of *W*_BF_.

In Fig 7A the blue and red shaded regions represent the strategies where the linear mixed model identified zero score advantage, respectively for the slow and fast stimulus conditions. The shaded regions in Fig 7A represent one standard error of the estimates. The positive slopes of the shaded regions in Fig 7A indicate that human actors traded off larger depth of computation (large *d*) for more frequent recalculation (small *r*). For example, at the low stimulus speed, strategy (*r* = 1, *d* = 4) was consistent with human behavior. To extend their depth of computation one level to *d* = 5 human actors could no longer recalculate their plan at every step, but only every 3-4 steps. Thus, that single extra level of depth came at the cost of being stuck with a potentially out-of-date plan 2-3 steps longer than when actors restricted their depth.

The tradeoff between *r* and *d* in the inferred human strategies suggests that their behavior was bounded by a limited amount of computational resources: a limited number of computations per step. To evaluate the potential paths across the stimulus, the actors had to perform a certain number of summations on every step. In Fig 7B we plot the number of summations required for a brute-force summation of all the possible paths across the stimulus. For a given depth *d*, there are 2^*d*^ paths, each of which requires *d* summations. Moreover, increasing recalculation period *r* implies that the actor performs 1/*r* fewer total summations. Thus, the average number of expected summations (which we call “brute-force workload” *W*_*BF*_) for strategy (*r*, *d*) is

$$\begin{array}{c} W_{BF}(r,d)=\frac{d+1}{r}2^{d}, \end{array}$$ (2)

which is plotted in Fig 7B as a map in (*r*, *d*). The black curves in Fig 7B are the contours of equal workload. The slopes of these contours reveal that the brute-force computation entails a tradeoff between *d* and *r*, similar to the tradeoff we observed in human behavior (Fig 7A). That is, human behavior was consistent with the brute-force search strategy under the limited amount of computations per step.

The iso-workload contours plotted in Fig 7B were obtained by numerically solving Eq 2. We selected the two iso-workload contours that matched the human strategies in Fig 7A at the center of their range of *r* values (copied there from Fig 7B). For the “fast” condition, the contour represents *W*_*BF*_ = 12 ± 2, and for the “slow” condition it represents *W*_*BF*_ = 48 ± 8. (The errors were propagated from the error in *d* at the center of the range of *r*). Remarkably, these contours make excellent approximations to our estimates of human strategies.

We also compared human strategies to an optimal computational algorithm. From the optimal computation (derived in section *Methods: Summation algorithms*), the average number of expected summations is the “efficient workload” *W*_*E*_ for strategy (*r*, *d*):

$$\begin{array}{c} W_{E}(r,d)=3dr-\frac{3}{2}r^{2}-\frac{1}{2}r+2d, \end{array}$$ (3)

mapped out in Fig 7C. The magnitudes of this map are lower than in the map of *W*_*BF*_ in Fig 7B. Notice that, for a given *d* in the map of *W*_*E*_, the smaller *r* can be easier to calculate than larger *r*. Since a smaller *r* would lead to a higher score, the actors using the efficient algorithm would tend to engage the smallest possible value of *r* = 1. The contours of constant workload *W*_*E*_ are characterized by both negative and positive slopes, which are inconsistent with our estimates of human strategies in Fig 7A. Similar to our analysis of the brute-force algorithm, we attempted to match the iso-workload contours of *W*_*E*_ to the human data. The gray contours shown in Fig 7A and 7C were chosen for the workloads of *W*_*E*_ = 9 ± 1 and *W*_*E*_ = 21 ± 1, for the “fast” and “slow” conditions, respectively.

This analysis supports the view that humans evaluated potential paths using a method similar to the comprehensive brute-force summation constrained by a finite workload. Further evidence for this somewhat unexpected result, that humans completely examine their choices up to a finite depth, comes from our analysis of disk utility below.

### Analysis of reaction times

**Fig 8:**
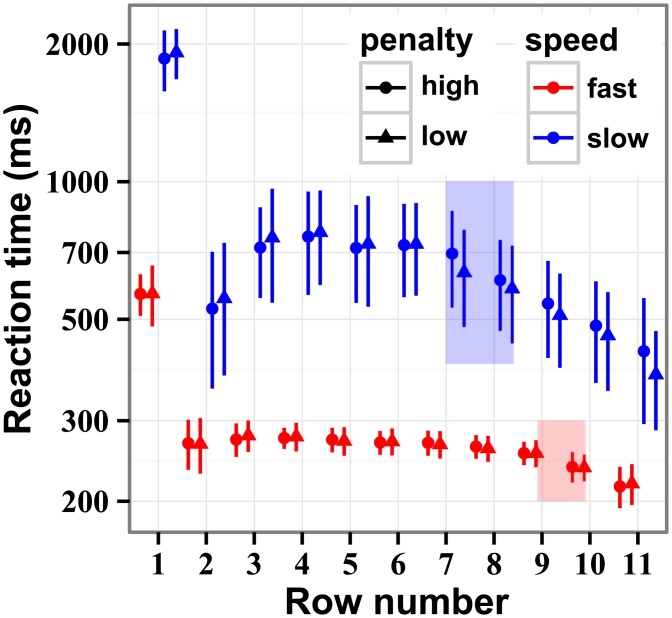
Reaction times averaged for all participants. The data are plotted separately for the speed and penalty conditions. The reaction times are markedly longer on the first stimulus row, just after the stimulus is revealed. Towards stimulus end, the reaction times drop off, reflecting the reduced amount of computation required for planning action over the reducing number of remaining rows. The shaded blue and red regions represent the rows on which the number of rows to stimulus end is equal to participant’s estimated depths of computation, where the approaching stimulus end is expected to have an effect on reaction times. Indeed, the reaction times start to drop at about the predicted rows. The data are averages of log reaction times (since the data were highly skewed) and the error bars are bootstrapped 95% confidence intervals.

Reaction time was defined as the time between subsequent choices (Fig 8). Reaction times followed a log-normal rule, and were log transformed for further analyses. The overall average reaction time was 991 ± 9 ms. Actors spent more time looking at the stimulus in the beginning of the trial. Reactions times were 771 ± 7 ms longer for the first choice (*χ* ^2^ (1) = 18.35, *p* = 1.8 × 10^−5^). Over the middle rows (rows 3–7) the reaction times were stable.

Toward stimulus end, the amount of computation could be reduced because of the diminishing number of remaining rows. The solid colored regions in Fig 8 indicate where the estimated depths of computation (described in the next section) are greater than the remaining number of rows. Indeed, it is expected that the reaction times should drop off when the computations become simpler. The approximately linear drop off of the subsequent reaction times is consistent with the notion that participants reduced the computation by a constant unit for each decrease in depth of computation.

Actors adapted their reaction time to the speed of presentation, but not to the penalty level. The reaction time for the low speed was 528 ± 4 ms longer than for the high speed (*χ* ^2^ (1) = 17.93, *p* = 2.3 × 10^−5^).

### Analysis of paths

**Fig 9:**
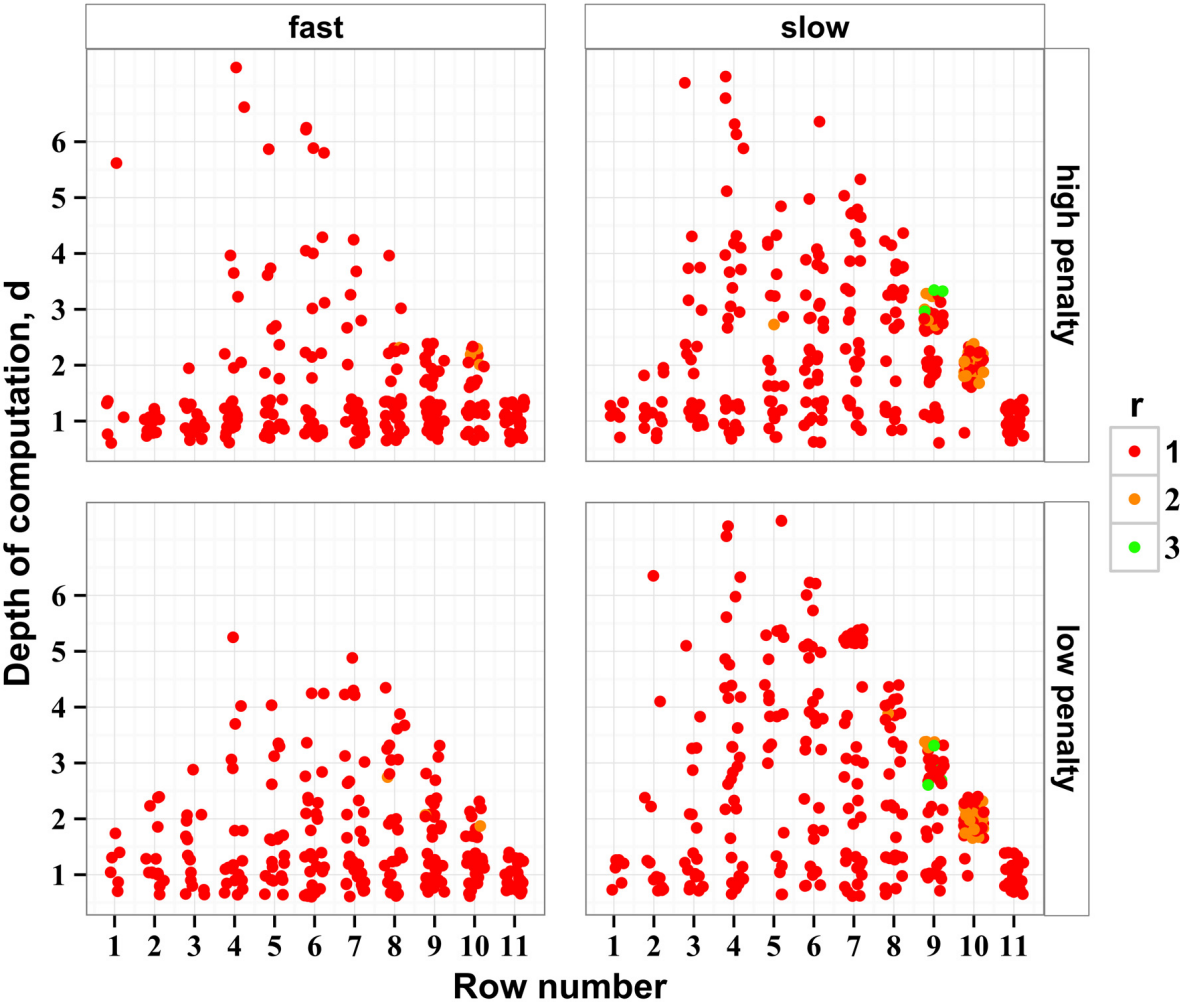
Depths of computation inferred from the maximally overlapping strategies for every row and for all stimuli. Each point represents one participant visiting one disk at the respective row at least ten times. The height of the point represents the depth of the most likely strategy found on the corresponding row. For clarity, the points are jittered near their corresponding values of *d* and row. On rows 1–3, the depths appear to be very low, reflecting a transition process following stimulus onset. The points form an upper envelope whose edge has a negative slope on the right side of every plot, reflecting the decreasing number of rows to stimulus end. The envelope is more salient in the plots for the low-speed condition, where the estimated depths of computation are higher than for the high-speed condition.

In the analysis of behavior presented in section *Analysis of scores* we made two assumptions: first, that participants used a consistent strategy across stimulus rows; second, that participants’ total score adequately represented their behavior over the entire stimulus. Here we relax these assumptions. Using a method of voting, we study how human choices varied across the rows. For every disk on every stimulus touched by a participant, we count the number of times their subsequent behavior overlapped with the trajectories selected by every possible ideal actor starting from that disk. Every overlap is counted as a “vote.” As before, the ideal actors are parameterized by depths of computation and recalculation periods (*r*, *d*). For all stimuli, for every participant, condition, and stimulus disk, and given a sufficient number of touches (as described in Methods), we view the strategy with the highest vote as an estimate of the participant’s strategy at that disk. The estimates form a map of likely strategies over the stimulus for every speed and penalty condition (Fig 9). Details of the voting algorithm are described in *Methods*.

To combine the estimates of strategies, we concentrated on the intermediate rows of stimuli, away from their beginning and end, where we found distinct boundary effects (Fig 9). On the first stimulus rows, the estimates of *d* were artificially diminished, reflecting properties of the of stimulus rather than human behavior. Indeed, the disks on rows 2 and 3 tended to be small, evident in Fig 3, which is why the scores accumulated by ideal and human actors on these rows were very low (Fig 5). In other words, the first three rows were not indicative of human behavior. They rather reflected a transitional behavior evoked by revealing the stimulus. On the last rows of the stimulus, *d* was limited by the remaining number of rows. An effect of this reduction is reflected in Fig 9 and manifested as a linear “envelope” on the right side of the plot, evident after about row 8. This pattern is more distinct in the low-speed condition, where the depths of computation were presumably larger. We find a stable behavior over rows 4–8. The recalculation period of *r* = 1 dominates this region. We fitted the data using a generalized linear mixed model with fixed effects of speed and penalty, and scalar random effects of participant, speed, and penalty by participant (chosen by testing models and selecting the smallest profiled deviance), assuming a Poisson distribution of depths. There was only a main effect of speed, where the high speed was on average 40 ± 20% faster than low speed (*χ* ^2^ (1) = 10.9, *p* = 0.00094). This is in agreement with the results of score comparisons presented in section *Analysis of scores* (*d* = 3.5 ± 1.5 for high speed and *d* = 5.0 ± 1.8 for low speed) taking into account the correction factor described in *Methods*.

Some of the ambiguity of the evidence calculation comes from the stimulus optimization algorithm because it is less reliable in separating the shorter paths. The paths from different depths of computation are separated from each other by the strategic placement of a large reward some distance ahead. For example, the paths resulting from a strategy characterized by *d* = 5 have six opportunities for a large disk to appear on the fifth row ahead, but a *d* = 1 strategy has only 2 such opportunities. Thus, the paths selected by smaller-depth strategies are misidentified more often than the paths selected by larger-depth strategies. It often happens that the step selected by a human overlaps with a step resulting from only one ideal planner, allowing for unambiguous identification of human strategies. Such lack of ambiguity was found in 326–382 cases per participant where human behavior overlapped with the ideal path from exactly one depth of computation. 54 ± 2% of the unique overlaps came in the slow condition and the rest in the fast condition (disregarding penalty conditions).

**Fig 10:**
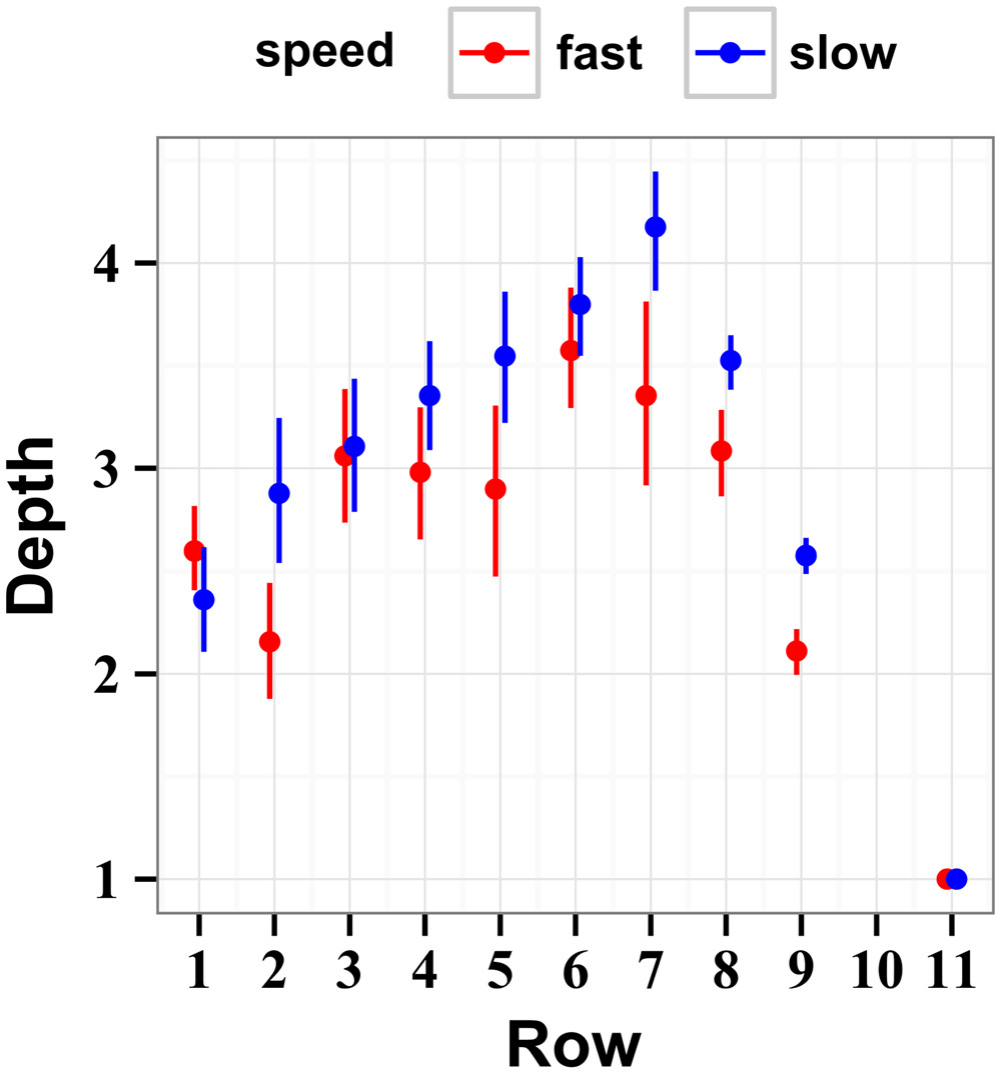
Depths of computation estimated using only unambiguous path overlaps. The unambiguous recovery of depth of computation *d* from human choices is achieved by taking into account only those choices that overlap with just one ideal path. (Row 10 had no such overlaps). The estimates of *d* based on the certain choices do not suffer from the artificially inflated evidence for *d* = 1 observed in Fig 9.

Result of this analysis are plotted in Fig 10, averaged across participants for each row. Similar to the POMDP and the brute-force policies, the depth of computation increases toward the middle section of the stimulus and then decreases where the participants are able to see the end of the stimulus. In the slow condition, the drop-off starts at row 8 (4 rows left to go), and in the fast condition the drop-off starts at row 9 (3 rows left to go), in agreement with results of the reaction time analysis. We built a linear mixed model using factors of row and speed and random effects of stimulus speed by participant and stimulus total score. The last row (where *d* = 1 is the only option) and row 10 (where there were no unique overlaps) were excluded from this analysis. We found a significant effect of interaction of speed and row (*χ* ^2^ (8) = 22.86, *p* = 0.0036) as well as main effects of speed (*χ* ^2^ (1) = 5.12, *p* = 0.024) and row (*χ* ^2^ (8) = 301, *p* < 1*e* − 15). The depth was an average of *d* = 3.3 ± 0.3 for the slow condition and *d* = 2.8 ± 0.4 for the fast condition.

### Analysis of utility

In the analyses above we made the assumption that actors were taking into account all disk sizes and that the subjective value (or utility) of disks of different sizes correctly represented their worth. Studies of human decision-making have shown that utility of expected events is generally different from their monetary or other objective worth. For example, humans tend to undervalue large-loss low-probability events [28], leading to demonstrably reduced utility of risky bargains. In contrast, temporally removed events have disproportionately small utility, a phenomenon called “temporal myopia” [29] or “discounting” in the reinforcement learning literature [30].

To measure the utility of different disk sizes, we asked whether participants attempted to simplify their task by employing the heuristics we call *seeking–the–large* and *avoiding–the–small*. In other words, we studied whether participants reduced the computational difficulty of the task by effectively merging certain disk sizes, for example by considering disks larger than a certain size as “large” and then trying to reach only those disks, while ignoring all the smaller disks. In case of seeking out the large disks, participants would increase the expected positive value of the largest disks as compared to smaller disks. And in case of avoiding the small disks, participants would make the expected small or negative values of the smallest disks even smaller or more negative (negative because of the penalty for missing a disk), and thus avoid the penalty for missing the small disks.

We simulated these heuristic strategies by reweighting the disks for the ideal actors. The ideal actors were forced to *seek the large* by making any disks smaller than a given size uniformly small. For example, when the ideal actor would have seen disks of sizes 64 + 64 to the left and of sizes 1 + 81 to the right, it would normally go left, to collect the larger score of 128. But a strategy of *seek–the–large* would move the actor to go right, to capture the 81 points. Reweighting the 64-point disk to 1 point induces the ideal actor into performing just such a strategy. Similarly, *avoid-the-small* is a strategy wherein actors try to avoid the penalties accrued on missing the smaller disks. Ideal actors were forced to avoid the small by making the disks larger than a given size very large. For example, an actor might see 1 + 81 to the left and 36 + 36 to the right, normally moving the actor to go left for a total of 82 points. But, a risk averse actor would go right to avoid the dangerous 1-point disk, at the cost of accumulating 10 fewer points. Reweighting larger disks to the very large value of 1000 points forces the ideal actor to behave this way, so that it sees 1 + 1000 to the left and 1000 + 1000 to the right, and chooses to go right. These heuristics are both related to the notion of “pruning” the decision trees. The avoid–the–small heuristic is equivalent to pruning in the sense that the former effectively ignores the branches of decision tree that contain small rewards, whereas the seek–the–large heuristic considers only those branches that contain large rewards.

**Fig 11:**
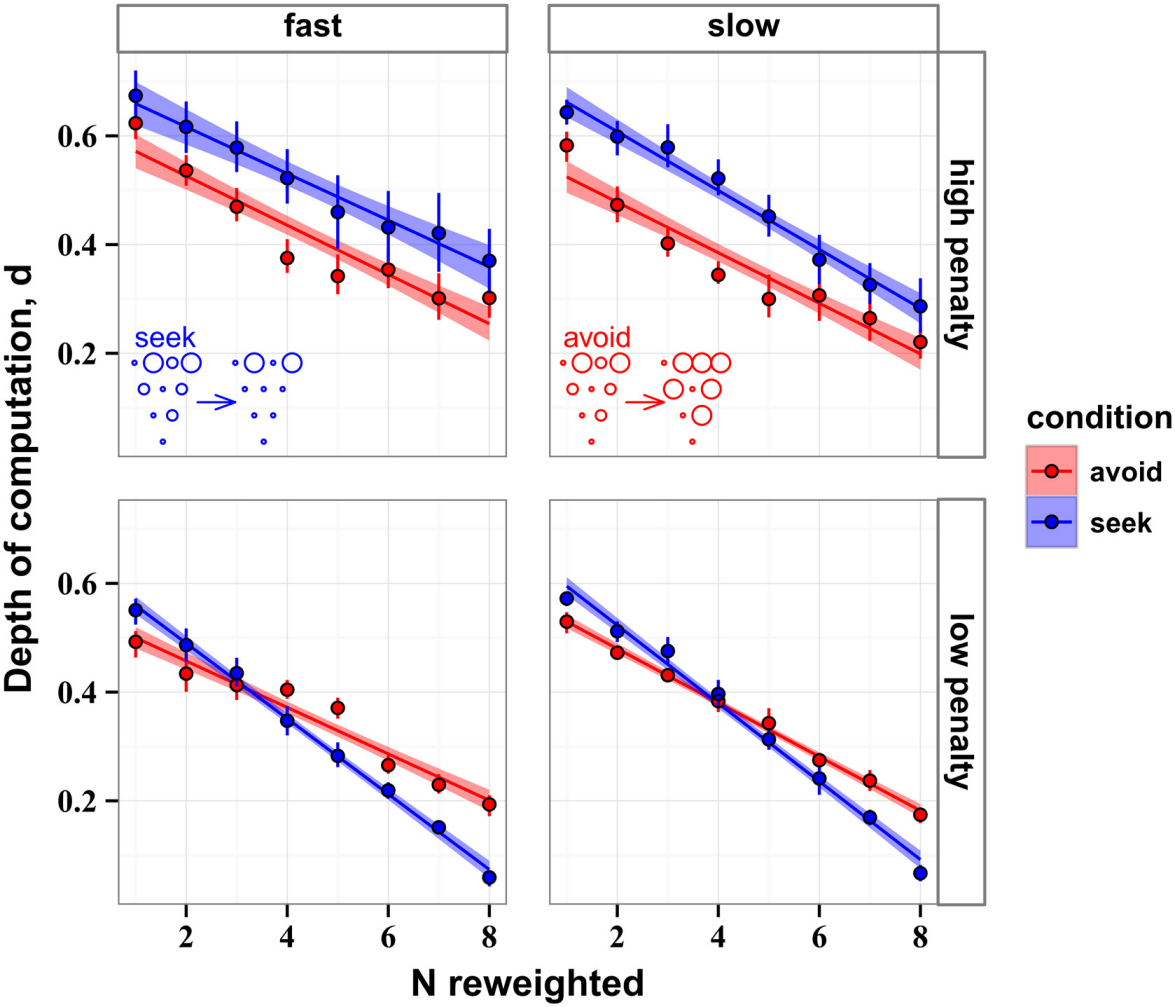
Results of the weighting analysis averaged over participants. Blue lines represent the effect of seeking out large disks and red lines represent the effect of avoiding small disks. The lines are the best fits to the data with the standard errors shaded. The points are the means with the 95% confidence intervals represented by the error bars. Top and bottom rows correspond to the conditions of high and low penalties of missing a disk, respectively. Left and right columns correspond to high and low speeds of the stimulus. The smaller values on the ordinate the larger the evidence that participants either sought out the largest disks (blue) or avoided the smaller disks (red). The fact that evidence is always positive indicates that participants never relied on these heuristics fully.

Results of the weighting analysis are displayed in Fig 11, averaged across all participants. The plots represent how much the evidence for different strategies used by actors (Eq 4) was changed by disk reweighting, using the different numbers of reweighted disks marked on the abscissa. (For avoid–the–small, one or two reweighed disks means that only the largest or only the two largest disks were reweighted, respectively; and for seek–the–large, one or two reweighed disks means that only the smallest or only the two smallest disks were reweighted, detailed in *Methods: Weighting analysis*). Zero “difference of evidence” would mean that the human actors behaved as if they either sought out the largest disks or avoided the smaller disks. One-sided t-test showed that the difference of evidence was positive for all conditions (*t* (5) > 7.47, *p* < 0.001). The deviations from zero for every number of reweighted disks in Fig 11 indicate that the disks “excluded” by weighting were in fact used to make decisions. Remarkably, participants behaved as if they took into account almost all disk sizes, rather than relying solely on the largest disks or avoiding the smaller disks. This result held for every participant, indicated by the small error bars in Fig 11.

When comparing between the low and high penalty conditions (i.e., between the top and bottom rows in Fig 11), we found that the difference of evidence was larger in high-penalty (0.5092 ± 0.0167) than low-penalty conditions (0.2128 ± 0.0068, *χ* ^2^ (1) = 17.5, *p* < 0.001). That is, the simple heuristics (avoid-the-small or seek-the-large) were of lower value when the penalty was large.

Interestingly, when penalty was high (top row in Fig 11), the difference of evidence for the seek-the-large strategy (blue lines) was on average 32.7% higher than for the avoid-the-small strategy (red lines). Statistically, this change appears as a significant interaction between penalty and stimulus speed (*χ* ^2^ (1) = 25.3, *p* = 4.9 × 10^−7^). This result indicates that humans tended to avoid small disks more when the penalty for missing disks was higher. In other words, as the penalty increased, avoiding the small disks was more important than seeking out the large disks.

### Comparison with partially observable Markov decision process

While score comparisons and voting procedures above yielded clear results, a potentially more powerful but data hungry approach relies on partially observable Markov processes (POMDP) [23, 24]. Fueled by the realization that such models can be written as Markov models in an associated belief space, standard methods have been developed for finding optimal sequences of actions in robotics [31, 32]. On the practical side, random sampling techniques were applied to finding optimal policies in this framework, which led to the development of Successive Approximations of the Reachable Space under Optimal Policies (SARSOP) algorithm [33, 34] that we use to illustrate this approach below.

**Fig 12:**
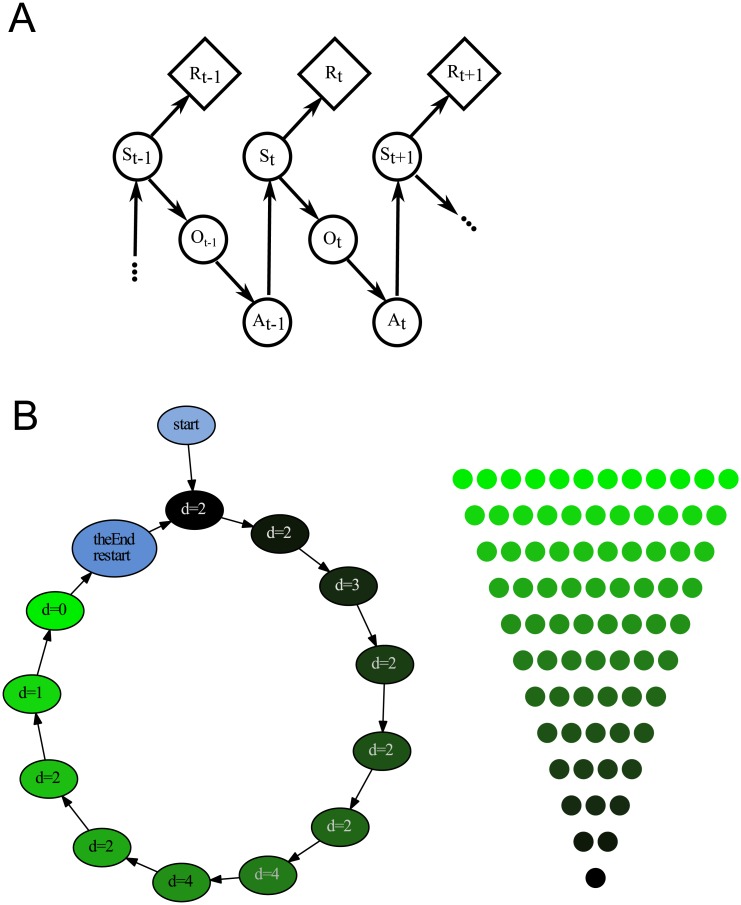
Partially observable Markov decision process (POMDP). (A) Elements of POMDP. Upon entering state *S*_*t*_ the actor receives reward *R*_*t*_ and observation *O*_*t*_. The observations are used to select action *A*_*t*_ that results in moving to state *S*_*t* + 1_. This iterative process repeats for multiple stimuli. (B) Results of a POMDP simulation. The nodes on the circular policy graph represent stimulus rows. At each node, the simulated actor chooses to look ahead for some distance *d* and translates the maximum score differential to the probability of stepping left or right. The simulated actor makes 11 such choices (for the 12 rows of the stimulus) and then starts the next trial using a stimulus randomly chosen (with uniform probability) from the set of all stimuli.

POMDP models have six components: “states” of the system, “rewards,” “transitions” between the states, “observations,” “actions,” and a “discount factor” (Fig 12A). Applied to our task, definitions of the states, rewards, observations, and transitions are immediately clear. The “states” are the locations in the stimulus (specific disks) and the “rewards” are the values gained by selecting individual disks. (A more detailed description of the POMDP model is given in *Methods: Model of partially observable Markov decision process section*). There is one additional state (labeled theEnd in Fig 12B) that separates trials and provides no reward. There are two kinds of “observations:” that a movement occurred and that a trial was restarted. The “transitions” are movements from disk to disk along the allowed edges and to a new stimulus.

The actions of moving left or right are determined probabilistically by a sigmoidal function of the maximum possible scores up to a given depth of computation. For example, using *d* = 2 in Fig 1, the simulated agent will observe a maximum score of 16 + 81 = 97 for a left choice and 64 + 16 = 80 for a right choice, yielding an expected maximum score difference of 17. The probability of taking the left choice is *P*_*L*_(17, *β*_2_, *η*_2_) where *P*_*L*_ is the sigmoid function with sensitivity parameter *β*_2_ and bias parameter *η*_2_ (explained in greater detail in *Methods: Model of partially observable Markov decision process*). In the binary task, the probability of taking the right choice is 1 − *P*_*L*_. Agent’s actions are notated dLook where the values of depth of computation *d* are defined on the interval from 0 to 5 steps (0 corresponds to the random behavior): 1Look, 2Look, etc.

An example of the optimal sequence of dLook actions is rendered in Fig 12B as a set of eleven sequential magnitudes of depths of computation *d*, one magnitude for each transition. (This illustration was derived using a subset of the stimuli employed in the experiments with human subjects). For a linearly increasing penalty (where the penalty for dLook is *d* points), assuming no left or right bias, a temperature parameter selected such that left is chosen 95% of the time for a score difference of 3 points (the smallest possible difference), and a discount 0.5, the optimal look ahead per row is (2, 2, 3, 2, 2, 2, 4, 4, 2, 2, 1). This is an illustration of the optimal strategy in which short depths are used early in the stimulus, and larger depths near the middle.

The POMDP approach offers powerful, flexible and general methods for solving problems of sequential decision making, but it relies on a specific parameterizations of the problem space. For example, the POMDP model of our task will require at least 19 free parameters, beginning with one discount factor and six assignments of penalties to depths of computation (arbitrarily assumed to be linear above). For each possible depth of computation, the sigmoid function is described by two parameters (*β*_*d*_ and *η*_*d*_ for *d* ∈ [0, 5]) for a total of 12 free parameters. What is more, the choice of the sigmoid function is arbitrary, although reasonable, and thus it implies an additional large set of parameters. Assigning at least 19 free parameters and selecting the sigmoid function afford an exceeding freedom. Fitting such a model to human data will require a correspondingly massive data set, assuming that at least ten subjects must be recruited per estimated parameter. Such large sets of data may become available by means of carefully designed consumer games or games entertained online by large groups of citizen scientists [35].

## Discussion

We devised a forced-choice task that challenged the human ability for prospective computation of reward. An array of pointing targets of different values formed a visual “decision tree” that scrolled down the screen at different speeds. Human participants (“actors”) selected a path over the stimulus to maximize the accumulated reward. We analyzed actor performance in terms of two factors: the horizon (“depth”) of computation, *d*, and recalculation period, *r*. Every combination of *d* and *r* defined a prospective *strategy*. To measure actor strategies we selected diagnostic stimuli and compared human behavior with the behavior of simulated (“ideal”) actors whose strategies were known and consistent.

One salient feature of human behavior revealed by this analysis is the tradeoff between the depth of computation and the recalculation period. The actors chose larger depths of computation at the cost of less frequent recalculation. Increasing the penalty for missing a target affected actor strategies weakly, but an increased time pressure caused actors to alter the tradeoff of *r* and *d*. Under low pressure (at low stimulus speed), actors used larger depths of computation *d*, but they only achieved the highest depth of *d* = 5 by increasing their recalculation period to *r* = 5 (i.e., not recalculating until they had to). When actors recalculated at every step, the depth of computation dropped to *d* = 4. Even under the light pressure, recalculating the prospective plan at every step prevented actors from looking as far ahead. The tradeoff was similar under high pressure (at high stimulus speed) when actors had lower depths of computation overall, but chose to either recalculate at every step with *d* = 2 or not recalculate until they had to with the higher depth *d* = 3. Thus, we observed a consistent tradeoff between strategy parameters *d* and *r*. The actors were able to extend their depth of computation *d* by one unit at the cost of sticking to the plan once it has been formed.

The present task can be solved by means of brute-force search that we implemented by simulating specialized ideal actors. Each ideal actor employed a single strategy and found the most rewarding path by considering every potential path under that strategy. Limitations of biological organisms may prevent them from rapidly performing the requisite computations. For example, consider a strategy with depth of computation *d* = 5 and recalculation period *r* = 1. In the exhaustive search under this strategy, up to 32 sets of five quantities have to be summed more than once on every step (which took human actors 558 ± 3 ms). Solving this task by means of dynamic programming requires computing a minimum of 30 independent sums for each choice (one for each edge on the decision tree). Explicit, deliberative summation at this speed is infeasible. And yet, our results indicate that our participants were able to perform the complete computation. Our results suggested that participants did not take advantage of simplifying heuristics, and that they took into account all the information within their current depth of computation. Indeed, a calculation of the total number of additions required to evaluate the paths (the “workload” illustrated in Fig 7) revealed that the average observed behavior had the same tradeoff of *r* and *d* as expected for the brute-force computation.

The finding of a tradeoff between depth of computation *d* and recalculation parameter *r* suggests that humans place high priority on the local computations. In the present rapid forced-choice task, the only means by which the actors increased their depth of computation was to delay the recalculation of the prospective plan. We found no evidence that the actors used satisficing heuristics to narrow their search. They did not seek out the largest disks nor did they avoid the smallest disks. This is surprising given the extensive evidence that in making complex decisions humans often use heuristics rather than perform optimal computations [1, 2, 36]. Studies of decision-making in “higher level” cognitive tasks found evidence that humans can use decision heuristics similar to the algorithms that “prune” decision trees. Such “pruning” is a form of the avoiding–the–small heuristic tested in this study, for which we found no evidence. Pruning is useful where the actor is unwilling to tolerate a large penalty, even when given the prospect of a large reward [3–5]. In our task, actors appeared to not prune decision trees, but take into account the entire set of prospective possibilities. The different results suggest that different decision strategies can be inspired by different stimuli. Our choice of stimuli allowed participants to rely on explicit visual information. This is in contrast to previous studies where the association of reward and stimulus had to be learned (e.g., [3]) or the stimuli were visually indistinct (e.g., [5]). Our results provide evidence that sensory information can rapidly inform prospective computations, perhaps because visual systems can process information from multiple retinal locations in parallel.

How our participants were able to perform the complete computations remains to be seen, but it is likely they employed the mechanisms specialized for representing the spatial layout of the visual scene. For example, posterior parietal cortex is often identified as the neural substrate for the short-term memory of visual scenes [37], encoding the spatial distribution of remembered visual stimuli [38], and possibly functioning as a buffer for holding information in a form accessible to decision-making [38, 39]. Posterior parietal cortex undoubtedly is just one part in a distributed system for such computations that possibly incorporates primary visual areas [39], dorsal striatum (for representing situation-action associations), ventral striatum (for representing stimulus value) [40], and prefrontal cortex. Prefrontal cortex is critically involved not only in working memory and sequencing, but also in set-shifting [13, 41, 42], which may be necessary for switching from one planning strategy to another. The computational capacity of such mechanisms limits the ability for prospective visual behavior, as does the speed with which these computations must be made.

The neuronal locus of prospective computations could be explored directly using various brain imaging and recording techniques. The limits of prospective behavior could be studied further by behavioral means, e.g., by representing the values of sequential locations symbolically (e.g., numerically) rather than by stimulus size, forcing the actors to rely on their higher-level (cognitive) faculties. By mixing symbolic and analogue representations of value (e.g., numerals and sizes), one could explore what visual information is used more readily in prospective tasks. Additional insights into human prospective computation could be gleaned using models designed in the general framework of partially observable Markov decision process (Fig 12), provided that the requisite massive data about human choices can be collected using “big data” approaches to sufficiently constrain such models.

In summary, we presented human actors with a novel task to traverse a branching multivalued stimulus and accumulate the maximal reward. Unlike the common task of selecting among a number of fixed rewards, the present task captured some of the complexity of natural behavior in the risky and dynamic world where ongoing decisions alter the landscape of future rewards. Using diagnostic stimuli, and by comparing human behavior with behavior of ideal actors, we uncovered several features of the planning strategies used by humans in face of this complexity. Human actors restricted their prospective computation to a horizon that varied with time pressure. They traded off two useful features of planning: large depth of the horizon vs. high frequency of recalculation. The tradeoff was predicted by an exhaustive search over all possible paths using a limited resource: a fixed number of computations. Humans did not appear to use simplifying heuristics, but they rather took into account all the information about the rewards. The computation included the smallest differences between stimulus parts within the horizon, even when making the fine distinctions yielded a very small (few percent) increment of the final reward.

## Methods

### Participants

Six participants (“actors”) including two authors (between 19 and 40 years of age, four males and two females) with no known neurological disorders participated in the study.

### Ethics statement

All participants signed a consent form approved by the Institutional Review Board of the University of California at San Diego (IRB #11615).

### Apparatus and procedure

The experiment was programmed in the Python language under Vizard 4.0 virtual reality platform (WorldViz LLC, Santa Barbara, CA). Participants sat in front of a 32” touchscreen (ET3239L, Elo Touchsystems) on which a triangular lattice of disks (a “stimulus”) scrolled down at a constant speed (Fig 1), viewed from a distance of 46 cm. The smallest disks had the radius of 5 mm, which was larger than the size of the average endpoint variance (4.8 ± 0.4 mm) of participants aiming at small targets, estimated in pilot experiments. The largest disk radius was 30.7 mm and the inter-disk distance was 62.7 mm.

The “trial” consisted of presentation of the entire stimulus. On every trial, the stimulus was initially hidden from view with only the bottom disk visible at screen center. Participants initiated the trial by touching the bottom disk. On this event, the portion of the stimulus fitting the screen became visible, and the stimulus started to scroll down revealing new stimulus rows (Fig 2), up to seven rows at a time. Participants made sequential binary choices by touching disks with a pen-like stylus. Every choice determined the range of future actions: only one of two disks in row *n* + 1 could be touched just above the disk previously touched in row *n*. As the participants progressed through the stimulus, the region over which they make their choices could drift away from the center of the screen (e.g., following a sequence of “left” choices). To compensate for this drift, the stimulus was moved horizontally (in addition to its continuous vertical scrolling) at a speed proportional to the distance of the last touch to the center of the screen.

The task was to maximize total score. Touching a disk incurred a score related to disk size: one point per unit of disk area ranging from 1 point for the smallest disks to 81 points for the largest disks. Disks had invisible circular buffers (radius 31 mm) around them that were larger than the largest disk and just barely covered the disk separation. A touch that missed the visible disk but hit the buffer was defined as a “miss.” This distinguished between motor performance errors (i.e., trying to hit a disk but missing it), and aiming for an invalid disk (e.g., a disk in the wrong row). Missing a disk incurred the loss of P (-1 or -50) points. The trial was aborted if participants did not make any choice for 10 seconds. Touching the screen above its midline had no effect, to encourage participants to look ahead of the presently engaged disks.

On every screen touch, distinct auditory tones informed participants whether they hit or missed a disk. Additionally, disks turned green or red to indicate the hits or misses, respectively. Background color indicated total score as follows. Dark red and dark blue were set to indicate respectively the maximal positive and maximal negative scores on every trial. The current background color was determined according to where the current cumulative score fell on the continuum between the two extremes. Additionally, three numerical values were displayed in the right bottom corner of the screen: the maximal possible score of the trial, the current cumulative score of the trial, and the cumulative score as the percentage of maximal possible total score over all completed trials.

There were four experimental conditions: two scrolling speeds of 63 and 189 mm/sec and two penalties of -1 and -50 points. In each condition, the same 100 different stimuli were presented, precalculated as described in section *Stimulus design* just below. The experiment consisted of two sessions, one session per day on two consecutive days. The session on the first day was used for training, to establish a stable level of performance following the initial learning. The order of the four conditions was counterbalanced across the participants.

### Stimulus design

We assumed that participants looked ahead for a variable number of rows (distance *d*) and selected a path across stimulus rows such as to maximize total score. They then followed the selected path for *r* steps before they recalculated the expected score and possibly chose a different path. We called parameters “depth of computation” *d* and “recalculation period” *r* (*r* ≤ *d*). Participant’s “strategy” was defined by parameter pair (*r*, *d*).

The stimuli were preselected such that different strategies by the participants would entail maximally different paths. The pre-selection was implemented using a Monte Carlo algorithm. For a single stimulus, first the algorithm found all optimal paths up to a depth of seven, starting from every disk of the stimulus. The optimal path was defined as the one for which the cumulative score was the largest among all possible paths. In score ties, all maximally scoring paths were stored. Then, a quantity called disk redundancy was computed for each stimulus disk: the number of strategies that predicted overlapping paths starting at that disk. Disk redundancy was then summed over every disk of the stimulus to generate a stimulus redundancy. Higher stimulus redundancy meant that more paths overlapped and less information could be gained from each choice by the participant. Stimulus redundancy was minimized using a Monte Carlo method. The initial condition was a random arrangement of disk sizes chosen uniformly from values 1, 4, 9, …, 81 (squared integers). Monte Carlo minimization randomly changed sizes of randomly selected disks and accepted the change either if it lowered the redundancy or with probability exp(−Δ*R*/*T*), where Δ*R* is the change of redundancy and *T* is the temperature parameter annealed from 1 to 0.5 to 0.25 to 0.125. The procedure ran for 1000/*T* steps at each magnitude of *T* (total of 15,000 per stimulus). This resulted in an average of 50 ± 7% (mean ± sd) smaller redundancy than the initial condition.

The stimuli were selected to minimize the overlap of paths generated by different strategies (without making an attempt to control the maximum score of the stimulus). The resulting distribution of disk sizes differed from stimulus to stimulus. The maximum score was in the range of 354 to 685, with a mean of 531 and a variance of 62. No stimulus design could achieve a full separation of the alternative paths, because the paths selected by different strategies inevitably overlap. For example, the strategy (*r*, *d*) = (2, 5) was equivalent to applying strategies (1, 5) and (1, 4) one after another. That is, looking ahead for five steps and taking the first two optimal steps is equivalent to looking ahead for five steps, taking only one step from the plan, and then looking ahead for four steps.

The relation between *d* and score is not necessarily linear. For example, we may define a sequence of strategies (a “policy”) for a number of subsequent rows. For example: “start with *d* = 5 on the first row, continue with *d* = 1 on the second row, *d* = 4 on the third row,” and so on. The policy can be represented as a tuple (5, 1, 4, …). The score of a policy over all the trees was represented as a fraction of the total possible score, and calculated for all possible policies on all trees. The calculation is simplified because of the hard boundary at the end of the tree: the actor should look to the end of the tree whenever possible, which is why the maximum policy will always end in (…, 5, 4, 3, 2, 1). Note that this calculation employed a brute-force algorithm since there are only 6^6^ = 46656 policies up to depth 5 (including *d* = 0 to represent a random choice) and 300 trees. The optimal policy is (1, 3, 2, 1, 1, 5, 5, 4, 3, 2, 1); it yields 93.0% of the maximally possible score, with the score variance of 0.07% across the of stimuli. In comparison, the maximum look–ahead policy (5, 5, 5, 5, 5, 5, 5, 4, 3, 2, 1) yields only 90.0% of the maximum score (variance 0.07%), and the worst scoring policy (5, 0, 5, 0, 0, 0, 5, 4, 3, 2, 1) yields 86.4% of the maximum (variance 0.07%).

### Summation algorithms

Here we evaluate the total number of summations required to evaluate paths for a given strategy (*r*, *d*). We consider two approaches: brute-force and efficient computation.

#### Brute-force computation

The algorithm directly performs *d* summations across all 2^*d*^ paths for a total of *d*2^*d*^ total summations. After accumulating the total score of each path, a comparison is made with the last path to decide if the new one is the optimum. Since we do not reuse the previously calculated sums, recalculation decreases the total number of sums only by a factor of 1/*r*. For example, if an actor recalculates after every two steps, the sums are only calculated half as often, and so on, for recalculating every third step. Thus, the total workload is

$$\begin{array}{c} W(r,d)=\frac{\left(d+1\right)}{r}2^{d}, \end{array}$$

which is Eq 2 in the main text, plotted in Fig 7B.

#### Efficient computation

This approach chooses the largest sum on every path while stepping through stimulus rows. This iterative dynamic programming approach distinguishes between the initial summation and later summations. For the initial phase, there are two sums to calculate on the first step, for the left edge (L) and the right edge (R). At the second step, there are four new sums to calculate, corresponding to paths LL, LR, RL, RR, but there are only three terminal disks on the corresponding row. Both LR or RL terminate at the same disk (in the middle), and we can decide which scores better with one comparison. Since the two paths end in the same spot, the only distinction between them is the score up to the end, and the larger scoring one will always be better. Similarly, for the third step, we have to do 6 additions (two choices for each of the three paths we kept) and decide on 4 to keep with 2 comparisons. This process is repeated for *d* steps with a total of $\sum_{i=1}^{d}2i=d\left(d+1\right)$ steps and $\sum_{i=1}^{d}i-1=d\left(d+1\right)/2-d$ comparisons. Assuming that comparison and summation operations have similar costs, the total number of operations to find the maximum sum for each end point at depth *d* is 3*d* (*d* + 1)/2 − *d*. Finally, we find the maximum of the remaining *d* paths which requires *d* comparisons. Thus, we are left with a total workload for the first step of

$$\begin{array}{c} W_{1}(d)=\frac{3}{2}d(d+1). \end{array}$$

We incorporate the recalculations on the later steps by noticing that we only add information for *d* − *r* rows onto the paths we already have. Specifically, there are three steps. First, subtract the tail (the path that was already traversed), from the *d* − *r* potential paths (we already know the number to subtract). Second, calculate the summations and comparisons as above for the *r* new rows. This sum is

$$\begin{array}{c} \begin{array}{cc} \sum_{i=d-r+1}^{d}3i-1 & =\sum_{i=1}^{d}(3i-1)-\sum_{i=1}^{d-r}(3i-1)\\ & =3dr-\frac{3}{2}r^{2}+\frac{1}{2}r. \end{array} \end{array}$$

Finally, we make *d* comparisons to find the best path. Thus, the total workload for the steps following the first step is

$$\begin{array}{c} W(r,d)=3dr-\frac{3}{2}r^{2}-\frac{1}{2}r+2d, \end{array}$$

which is Eq 3 in the main text, plotted in Fig 7C.

### Model of partially observable Markov decision process

We define a partially observable Markov process following the standard approach in terms of states, actions, observations, rewards, and a discount factor [23, 24]. The states correspond to all the disks on all stimuli, 12 × 13/2 = 78 disks per stimulus. Since the simulations of all 100 stimuli were not feasible, we used a random subsample of 20 stimuli. In addition to stimulus disks, there is an additional endNode that represents the step from stimulus to stimulus. There are two types of actions: lookD and restart. The lookD action uses a depth of computation *d* to calculate the maximum possible score for the next *d* steps given that the next step is to the left or the right. The score difference between choosing left and right (for a given *d*) is then mapped to a probability to take the left or right choice on the next step. Defining $s_{L}^{d}$ to be the maximum score given a left choice first, and $s_{R}^{d}$ given right first, the probability that the simulated actor goes left is modeled as a sigmoid function:

$$\begin{array}{c} P_{L}(s_{L}^{d}-s_{R}^{d},\beta_{d},\eta_{d})=1-\frac{1}{1+\exp[\beta_{d}(s_{R}^{d}-s_{L}^{d})-\eta_{d}]}. \end{array}$$

Parameter *β*_*d*_ controls the sensitivity (or “temperature”) of the sigmoid for each depth *d*: larger *β*_*d*_ means that any small advantage of the score to the left over score to the right will lead to choosing the left. Parameter *η*_*d*_ controls the bias of the model: larger *η*_*d*_ yields an advantage to making the left choice over the right choice even if the right score would have scored higher. For present simulations, we used *β*_*d*_ = 1 and *η*_*d*_ = 0, reflecting highly sensitive choices (in which actors choose the better scoring direction 95% of the time) and assuming no bias. The probability of moving right is one minus the probability of moving left.

The transition matrix for the POMDP is a sparse matrix for each action of a size equal to the total number of disks plus one for the endNode. Nonzero elements of the transition matrix occur when there is an allowed transitions between disks, an edge on the stimulus for the lookD action, or one of the starting disks for the restart action. The only transition allowed at terminal disks (on the last row of the stimulus) is to go to the endNode. The value of the nonzero elements for lookD transition matrices is the probability *P*_*L*_ to take the left choice and *P*_*R*_ = 1 − *P*_*L*_ to take the right choice. From the endNode the model goes to any of the start disks of the stimuli (the first row of the stimulus) with equal probability.

The model has two possible observations: move after a lookD action whenever a movement occurred, or restart when leaving the terminal node. The discount factor *γ* was a free parameter (we used *γ* = 0.5). The model was created using a custom C++ code which read in the preselected stimuli and generated files in the .pomdp format as described at www.pomdp.org. The POMDP solution was found using the SARSOP algorithm implemented in APPL (bigbird.comp.nus.edu.sg/pmwiki/farm/appl/) with standard options [33]. The policy graph was derived from the POMDP solution using the tools available from APPL to generate GraphViz formatted files (which we then edited for readability).

### Analysis of paths

The analysis of score presented in the previous section was complemented by an analysis of the paths selected by individual participants. We compared human choices on every stimulus disk with the choices predicted by every ideal actor. Using a custom C/C++ code, we first iterated through every disk of every stimulus. A table was assigned to every disk that held the optimum path (or paths in the rare case of a tie) starting at that disk for a given strategy (*r*, *d*), which is a sequence of left-right choices that maximize the total reward over the next *d* steps. (We could implement this brute-force approach because the stimuli had a limited size, but the efficient algorithm presented above could also be used). Only the first *r* steps were stored. Next, a participant’s path through the stimulus was read in, and the overlap was recorded with every strategy for every disk touched by the participant. The overlaps were coded as either 0 or 1. For example, to count as one overlap, strategy (2, 5) required that participants matched on two upcoming choices. This analysis yielded a large table that listed every touch by every participant and also how human choices compared with choices by ideal actors. The table was then analyzed using a custom R scripts for statistical tests as described below.

To validate the overlap method, we simulated 100,000 ideal actors with randomly chosen policies. The policies had a maximum *d* = 5 and fixed *r* = 1. The ideal actors were run through the stimuli (each actor went through one randomly selected stimulus), and we attempted to recover their *d* using the path overlap algorithm. The value of *d* recovered from the path algorithm was linearly related to the average *d*, but systematically underestimated the average *d* by a factor of 0.5 ± 0.1. The distribution of *d* from the path overlap algorithm was heavily skewed toward small *d*. The same randomized procedure was used to validate the score-advantage approach to estimating the average depth of computation where there was very little systematic bias (see Fig 5B).

### Weighting analysis

We analyzed human behavior in terms of how often participants’ paths overlapped with the paths selected by ideal actors for different strategies (*r*, *d*). For each disk of the path, all optimal paths up to depth *d*_max_ were calculated (usually *d*_max_ = 7). Then we compared the “measured” path selected by a human actor to the optimal paths by different ideal actors. If the measured path overlapped with the optimal path by an (*r*, *d*) actor, we incremented the vote counter for that ideal actor: *V*_*s*_ (*r*, *d*) = *V*_*s*_ (*r*, *d*) + 1. The result was an unnormalized measure of performance which we called “evidence.” We used this measure to compare human behavior with every strategy (*r*, *d*). To take into account the variability of individual stimuli, we calculated *V*_*R*_ (*r*, *d*) for *N* random paths through the stimulus. We found that *N* = 100 was sufficient to make the sampling error small. We normalized evidence by computing the ratio of these quantities:

$$\begin{array}{c} E(r,d)=\frac{V_{s}\left(r,d\right)}{\langle V_{R}\left(r,d\right)\rangle }, \end{array}$$ (4)

where the angle brackets indicate the average over *N* selections of *V*_*R*_. In effect, the lower values of *E* (*r*, *d*) correspond to the lower evidence for strategy (*r*, *d*). Values of *E* (*r*, *d*) ranged from 0 to 2, where *E* (*r*, *d*) = 0 indicated perfect avoidance of strategy (*r*, *d*), *E* (*r*, *d*) = 1 indicated a random behavior (a random choice of steps), and *E* (*r*, *d*) = 2 indicated a perfect overlap of the measured behavior with the behavior predicted for strategy (*r*, *d*). The maximal value of *E* (*r*, *d*) is 2 because there were two choices per step and thus the random planner would overlap with the optimal path half the time). The lower bound is strict but not necessarily attainable in the rare case where all paths from a disk follow the same strategy, i.e., in the presence of ties. The upper bound is not strict, but is subject to fluctuations in the sampling by the random planner. No measurements larger than two were observed. Importantly, results of the computation of evidence cannot be described by averaging (they do not self-average). That is, there exist many cases, such as *E* (1, 1) = 2 and *E* (1, 3) = 2 while *E* (1, 2) = 0, where depths 1 and 3 are used and depth 2 is never used. Thus, high evidence for depths 1 and 3 does not imply an average depth of 2. The measure of evidence can only be used for comparison between planners.

We studied how participants assigned values to different disk sizes using a reweighting analysis. We asked whether they sought out the largest disks or they avoided the smallest disks. The test of whether participants sought out the largest disks, consisted of several steps. First, the smallest eight disk sizes were changed (“reweighted”) to 1 point, such that all disks had the values of either 1 or 81 points (illustrated in Fig 11 in the top-left inset). The above measure of evidence was then calculated for the reweighted stimulus. Since only the largest disks were worth extra points, the ideal actors sought them out, and thus an increased evidence for the weighted stimuli would indicate a tendency of participants to seek out the largest disks. The process was repeated for keeping only the two largest disks, such that all disks had worth of 1, 64, or 81 points, then for the three largest disks, etc. We measured the RMS deviation of the evidence averaged over all strategies for each reweighting. The number of disks weighted to 1 where the RMS deviation approached zero indicated that the participants distinguished at most that many disks, i.e., if the RMS deviation was nonzero for weighting the two largest disks, then participants distinguished at least disks of size 1, 4, 9, …, 49 as having distinct values.

Similarly, we studied whether participants avoided the smallest disks. We reweighted the largest disks to a large value (1000 points) so that ideal actors would only avoid the smallest disk size. The evidences from the weighted and original stimuli were compared as above, and RMS deviation was calculated. Non–zero deviation when the *N* largest disks were reweighted meant that participants distinguished at least the 9 − *N* largest disks.

### Statistical tests

Linear mixed models were fit using maximum likelihood in R version 3.0.1 using the lmer package (version 1.0-6, http://lme4.r-forge.r-project.org/). Statistical tests were based on the *χ* ^2^ tests of the change in profiled deviance of models with and with out various terms [43]. The random effects were first found on the data set by examining combinations of the main factors and comparing their profiled deviance. For the score advantage calculation, a random effect of the stimulus nested within participant was used to model the participant and stimulus variability. For the RMS deviations of weight, random effects were penalty nested within participant. Then, with fixed random effects, the main effects were added sequentially starting with null model and adding terms by order. Thus, if there were two potential fixed effects A and B, the significance of, e.g., A would be calculated by comparing the profiled deviance of a model including just a linear effect in B with a more encompassing model including effects of A plus B. Additionally, to estimate the significance of an interaction, a model with just linear effects, A plus B, was compared to a full model including linear effects and the interaction term.
